# Controlling the femtosecond laser-driven transformation of dicyclopentadiene into cyclopentadiene

**DOI:** 10.1016/j.cplett.2012.10.054

**Published:** 2013-02-12

**Authors:** Tapas Goswami, Dipak K. Das, Debabrata Goswami

**Affiliations:** Department of Chemistry, Indian Institute of Technology, Kanpur 208016, India

## Abstract

Dynamics of the chemical transformation of dicyclopentadiene into cyclopentadiene in a supersonic molecular beam is elucidated using femtosecond time-resolved degenerate pump–probe mass spectrometry. Control of this ultrafast chemical reaction is achieved by using linearly chirped frequency modulated pulses. We show that negatively chirped femtosecond laser pulses enhance the cyclopentadiene photoproduct yield by an order of magnitude as compared to that of the unmodulated or the positively chirped pulses. This demonstrates that the phase structure of femtosecond laser pulse plays an important role in determining the outcome of a chemical reaction.

## Graphical abstract

**Graphical abstract:**

Control of a femtosecond chemical transformation of dicyclopentadiene into cyclopentadiene in a supersonic molecular beam is achieved by using linearly chirped frequency modulated pulses, indicating that the phase structure of the laser pulse plays an important role in determining the reaction outcome.

**Figure:**
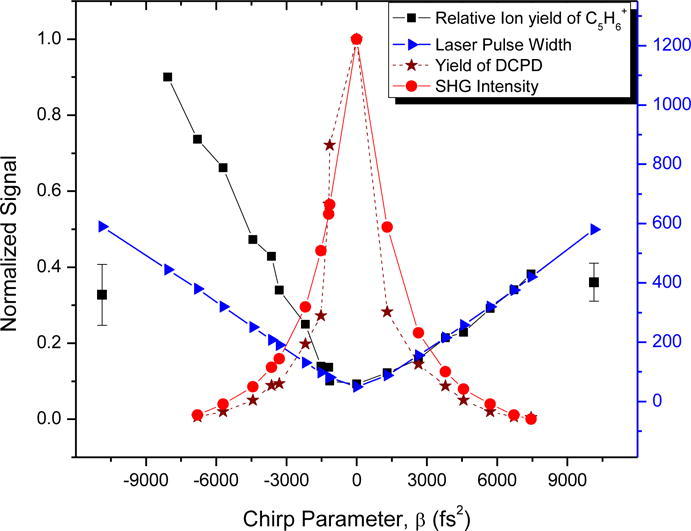

**Highlights:**

► Reporting the timescale of a femtosecond decylization reaction. ► Controlling the decyclization reaction through femtosecond chirped pulses. ► Distinguishing the reaction process from the fragment ionization process.

## 1 Introduction

Over the past decade, photo-fragmentation control has been illustrated for quite a few systems through the detection and interpretation of patterns in molecular fragmentation using an adaptive approach of ultrafast laser pulse shaping techniques [1], [2], [3], [4], [5], [6], [7]. Often, however, the final pulse shapes in such adaptive approaches were not found to be globally optimized [2], [3], [8], [9]. On the other hand, use of a simpler pulse modulation approach, e.g., the linear frequency modulation (chirping) resulted in more straightforward photochemical control and, as such, were defined as the chemistry where laser is used as a photonic reagent [10], [11]. The first experimental demonstration of control using linearly chirped pulses was made in the early 1990s [12], [13], which was followed by several attempts to control the yield of chemical reactions [14], [15]. Our recent work has shown that the laser induced molecular fragmentation of *n*-propyl benzene exhibits sensitive dependence on the linear chirp of femtosecond laser pulses [16]. Control of fragmentation and chemical reactions with simple linear chirped pulses as a control scheme has become an active field of research in recent years [9], [17], [18], [19], [20], [21].

**Figure 1:**
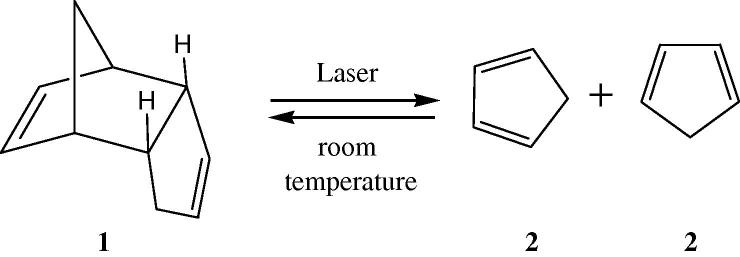
Photo-fragmentation schematic of dicyclopentadiene (**1**) as it forms two molecules of cyclopentadiene (**2**), which in turn dimerizes to **(1)**.

Real-time study of photochemical cyclization and decyclization processes has been of immense interest [22], [23], [24], [25], however, an attempt to control such processes is yet to be reported. We present here our results on the control of the photochemical reaction of dicyclopentadiene. We specifically explore the effect of chirping femtosecond laser pulses on the control of the conversion of 4,7-methano-3a,4,7,7a-tetrahydroindene (i.e., dicyclopentadiene or DCPD). As shown in the reaction schematic (Figure 1), DCPD is also the product of cyclic addition of two cyclopentadiene (CPD) molecules.

## 2 Experimental

We use a Ti:Sapphire multipass amplifier (Odin, Quantronix Inc.), which operates at 800 nm with 50 fs FWHM pulses at 1 kHz having energy of ∼1 mJ/pulse. Linearly frequency modulated ultrafast laser pulses (referred as chirped pulses) were generated from our suitably modified compressor setup for the amplified laser system. As we increase the spacing between the compressor gratings relative to the optimum position for minimum pulse duration of 50 fs, we generate a pulse whose leading edge is bluer than the trailing edge (negative chirping). Conversely, by decreasing the inter-grating distance, we obtain a pulse whose leading edge is redder than the trailing edge (positive chirping). Pulse durations for transform-limited pulse were measured using a homemade field autocorrelator. The negatively and positively chirped pulses were further characterized by the second harmonic frequency resolved optical gating (SHG-FROG) technique using a 0.1 mm type-1 BBO (beta barium borate) crystal before coupling the laser into the molecular beam chamber. DCPD sample at room temperature was used without further purification (98%; Sigma Aldrich) and was seeded in helium at 2 atm backing pressure. A pulsed valve (Series 9, General Valve) operating at 10 Hz repetition rate with 0.5 mm diameter was used to generate the supersonically expanded molecular beam of DCPD, which was allowed to interact with the laser beam at the center of a time of flight chamber. A skimmer (Beam Dynamics Inc.) of 1 mm diameter was used to collimate the supersonic molecular beam before introducing it into the laser interaction region. The pressure in the interaction chamber during the experiment was kept at 10^−7^ torr (base pressure of 5 × 10^−9^ torr). The laser beam was focused onto the molecular beam with a 50 cm focal length lens. The polarization of the laser was kept horizontal as it enters the mass spectrometer and is perpendicular to ion collection optics. Synchronization of the 10 Hz molecular beam pulses of 100 μs pulse duration with the 1 kHz femtosecond laser pulses is achieved by a homemade countdown circuitry with delay generator. The mass spectra were recorded with a Wiley–McLaren type linear time of flight mass spectrometer. The fragment ions are detected using an 18 mm dual micro-channel plate (MCP) detector coupled to a 1 GHz digital oscilloscope (LeCroy 6100A). Mass spectra were typically averaged over 200 laser shots.

**Figure 2:**
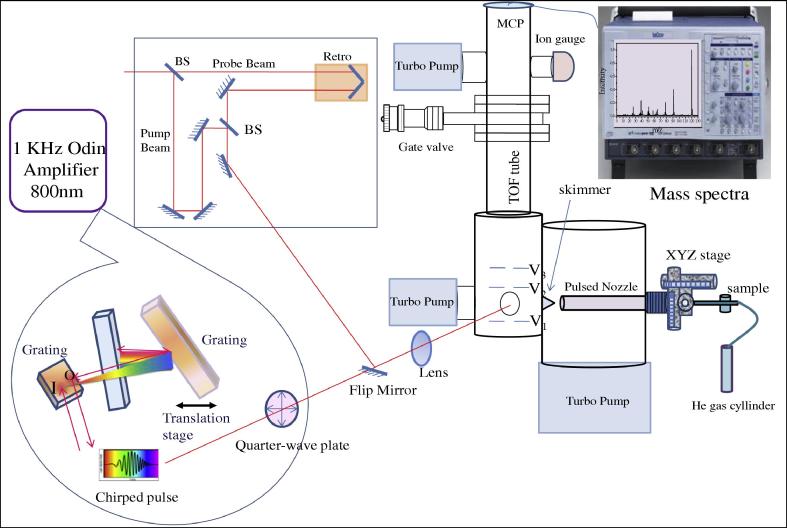
Schematic experimental setup showing the amplified chirped laser interaction with the supersonic molecular beam chamber.

The incident laser power intensity was varied by variable neutral density filter keeping the beam focusing geometry and beam diameter fixed for the study of laser power dependence on the ion yield. Degenerate femtosecond pump–probe spectroscopy at 800 nm was used to measure the ultrafast dynamics of the photo-fragmentation reaction of DCPD. Specifically, for the collinear degenerate pump–probe study, the laser beam was split to provide pump and probe beam by an ultrafast 70:30 beamsplitter. The weaker probe beam was made to travel through a computer controlled motorized delay stage and was then spatially combined with the pump beam by an another 70:30 ultrafast beamsplitter and were focused onto the supersonic molecular beam containing a linear time of flight mass spectrometer. To ensure that the mass spectroscopic contribution is only from the probe beam, the pump beam intensity was kept smaller than the probe beam. The one-color transient ionization spectra, i.e., the ion signal for a given mass in the TOF spectra dependent upon the time delay between the pump and the probe laser pulses were measured by 1 GHz oscilloscope interfaced with computer. Both photochemical control and one-color pump–probe study were performed using our versatile experimental scheme discussed here (Figure 2). The zero delay between the transform-limited pump and probe pulse was determined by field autocorrelation measurement [26] just before the interaction chamber.

## 3 Results and discussions

**Figure 3:**
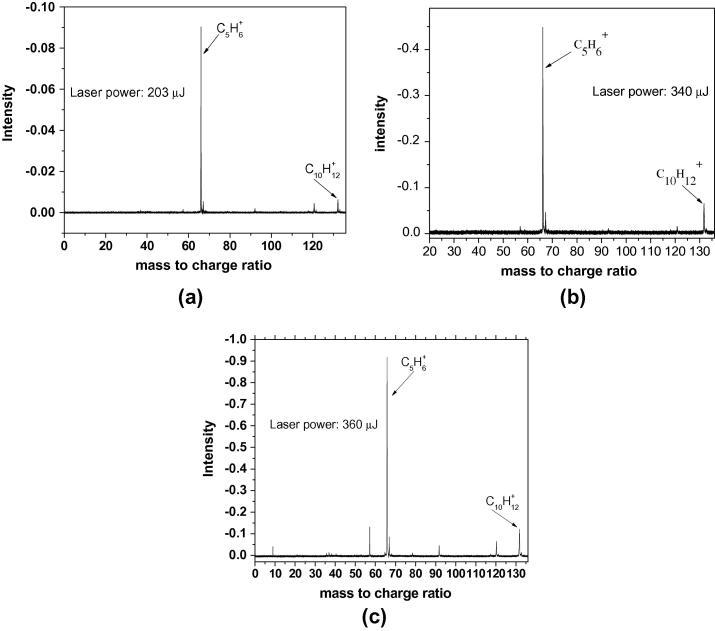
Mass spectra of dicyclopentadiene with 50 fs laser pulse showing the major two fragments: parent ion and cyclopentadiene ion collected at laser intensities of (a) 203 μJ/pulse, (b) 340 μJ/pulse, and (c) 360 μJ/pulse.

**Figure 4:**
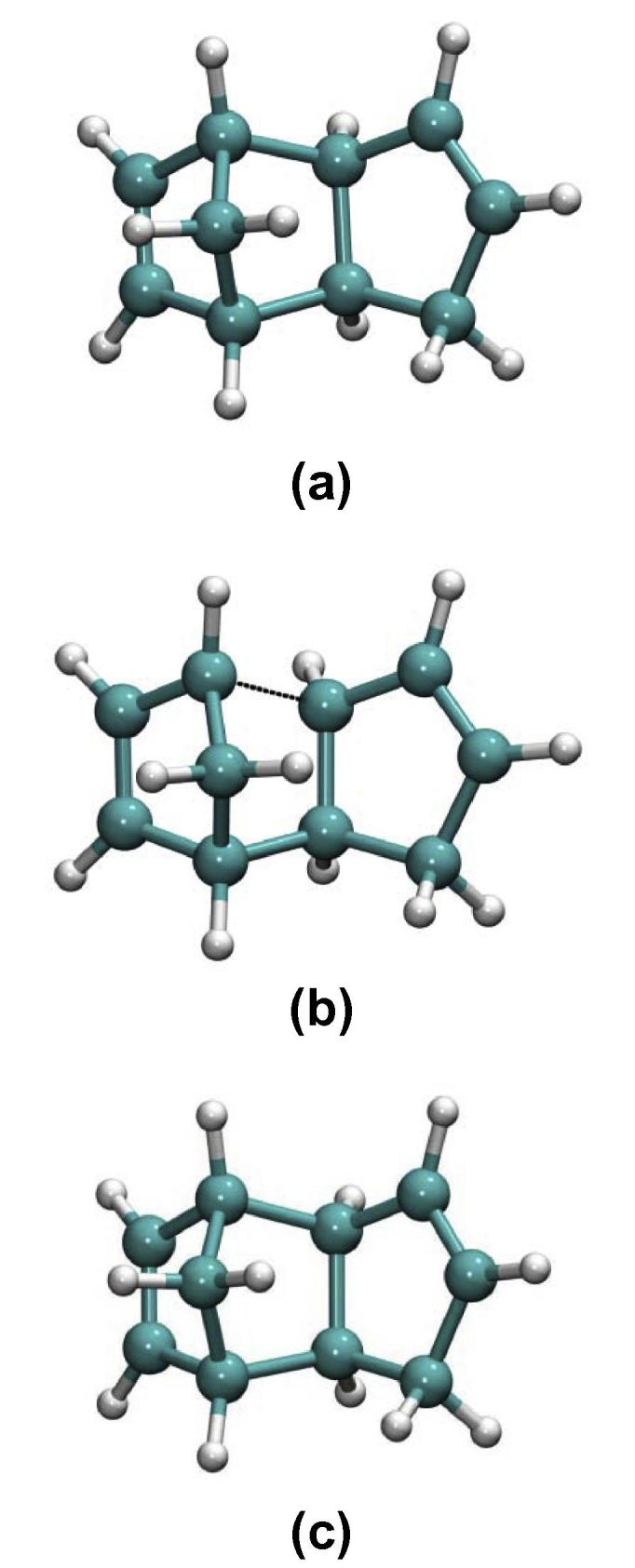
Optimized structure of (a) neutral, (b) DCPD^+^ (c) DCPD^2+^ using DFT (B3LYP/6-31G∗∗).

**Figure 5:**
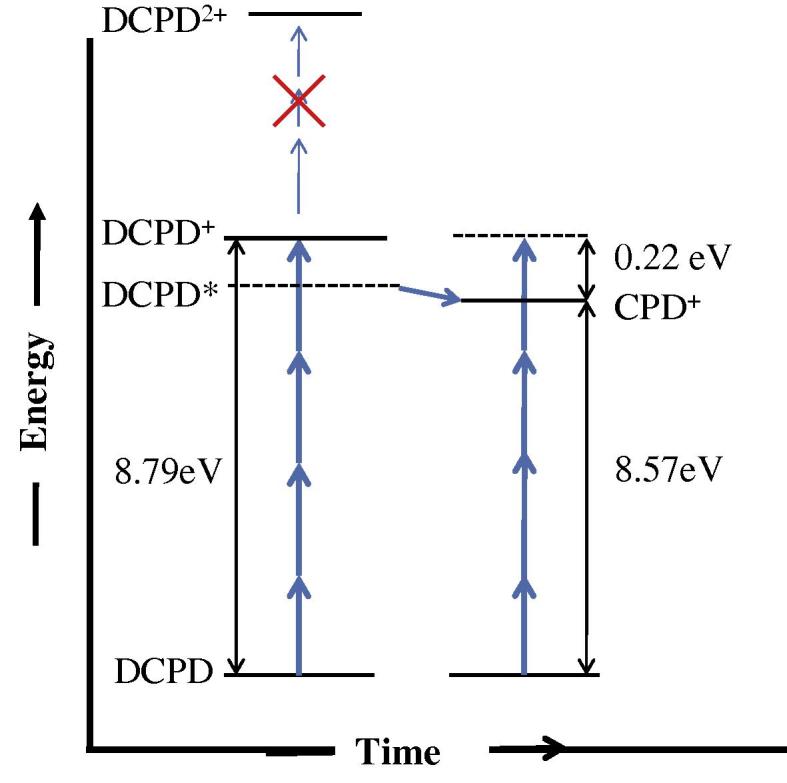
Schematic representation for the fragmentation of DCPD^+^.

The mass spectra of DCPD with 50 fs laser pulse having Gaussian temporal amplitude without any additional modulation in time, frequency or phase, i.e., a transform-limited pulse at three representative energies of 203, 340 and 360 μJ/pulse, respectively, is shown in Figure 3. The threshold laser energy (*I*_Th_) that we needed for detecting any mass spectroscopic signal was 180 μJ/pulse and the detected mass spectra was always dominated by two fragments having *m*/*z* values of 66 and 132. At 360 μJ/pulse energy and beyond, multiple mass fragmentations occur. In these mass spectra of the starting molecule DCPD, we assign the fragment with *m*/*z* of 66 to C_5_H_6_^+^ and that of 132 to C_10_H_12_^+^ (Figure 3). Another possibility of assignment of the peak corresponding to *m*/*z* = 66 could have been the formation of DCPD^2+^. However, we use density functional theory (DFT) methodology (B3LYP/6-31 G∗∗) to optimize the molecular geometries of the neutral, DCPD^+^ and DCPD^2+^ (Figure 4), and we find that the formation of DCPD^2+^ occurs at much higher energy than DCPD^+^ and C_5_H_6_^+^ (Figure 5). Thus, in our case DCPD^2+^ formation is very improbable, as the ionization potential for DCPD^2+^ is very high (∼20.8182 eV) as compared to that of the observed DCPD^+^ (8.79 eV) and C_5_H_6_^+^ (8.57 eV). Such energetic consideration further makes the *m*/*z* = 66 signal to correspond to C_5_H_6_^+^ since it is ∼8 times larger than that of the *m*/*z* = 132 (Figure 4). Also, the two carbon–carbon single bond dissociation energy needed for the inter-conversion of DCPD to CPD is less than 7.5 eV, which is also much lower than the ionization energy of DCPD or CPD. Thus, at *I*_Th_ = 180 μJ/pulse for ion-detection of the TOF mass-spectrometer, as soon as DCPD signal is formed, its conversion to CPD is automatically implied and this is evident from our experimental data.

**Figure 6:**
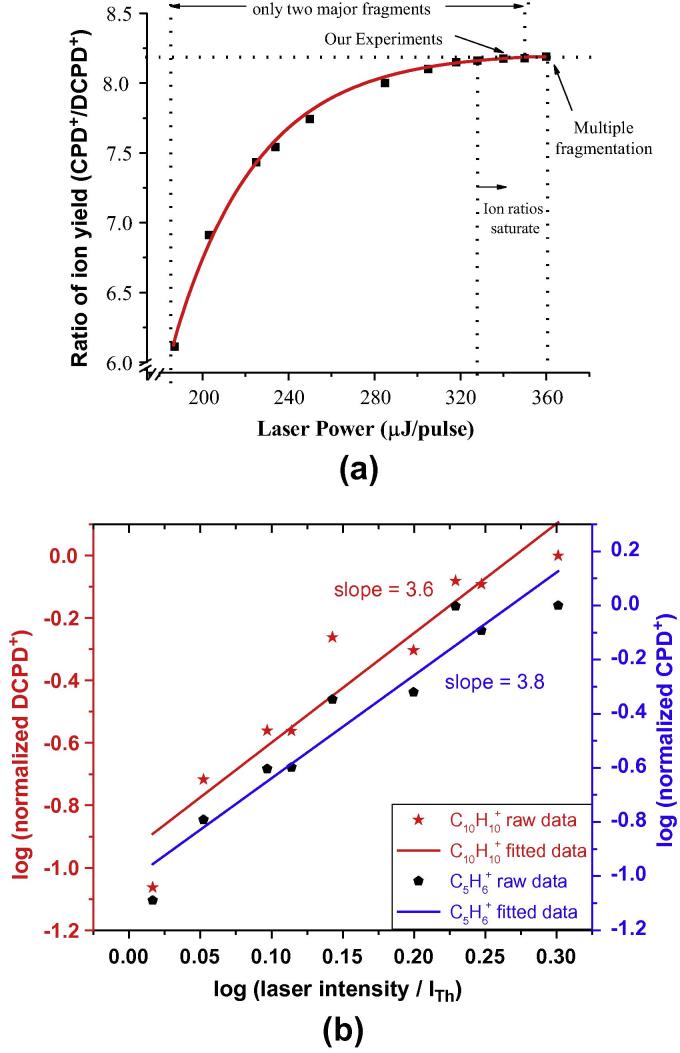
(a) Laser intensity dependence of the ratio of the cyclopentadiene yield to the parent dicyclopentadiene ion. (b) Laser intensity dependence of the parent dicyclopentadiene ion as well as the cyclopentadiene yield.

Before attempting the control of any chemical reaction, certain rudimentary knowledge of temporal dynamics of the system under study is essential. To determine the order of the multiphoton process, we studied the laser power dependence on the ion yield at fixed beam diameter and focusing geometry by varying the incident laser intensities (with a variable neutral density filter) between 180 and 360 μJ/pulse. As mentioned earlier, this intensity region is chosen as it extends from *I*_Th_ to the point just below multiple ion formation (Figure 3). A plot of the ratio of the CPD to DCPD ions with the laser intensity (Figure 6a) shows that this ratio undergoes an exponential growth followed by saturation. Log–log plot of intensity (normalized with respect to *I*_Th_) versus normalized ion yields of the ions is linear (Figure 6b) with a slope indicating that the reaction involves 4-photon transition with 800 nm laser and also confirms that all our experiments are in the regime of above saturation threshold intensity. This is in agreement with the one-photon studies at 200 nm as reported previously [27], however, for large aromatic polyatomic molecules, multiphoton ionization can occur below the ionization potential of the molecules [28]. Recently Scarborough et al. [29] have confirmed that the order of molecular fragmentation mainly depends upon the applied laser intensity. According to these studies, laser intensities that are sufficiently high but are still below the saturation ionization require fewer photons for ionization in comparison to the low intensity regime, which is in agreement with our experimental evidence of lower order of ionization. For such experiments, high signal-to-noise ratio is achieved. Control of chemical reaction will have practical application when such intensities are used, as sufficient yield and robustness of the ion signal would be achieved [21]. We also confirmed that changes in the laser polarization from linear to circular does not affect the pattern of the mass spectra, i.e., the relative ion yields remains roughly the same: the circular polarization only suppresses the ion yield.

**Figure 7:**
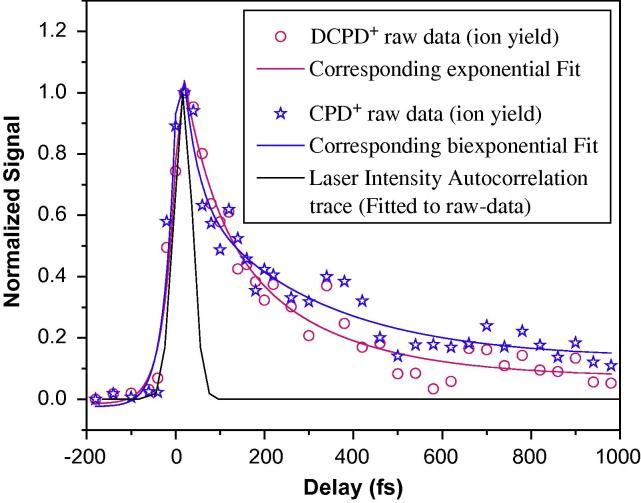
Degenerate pump–probe transient spectra at 800 nm of (a) the parent ion (C_10_H_12_^+^) and (b) the product ion (C_5_H_6_^+^) showing embedded wavepacket dynamics. (c) A comparison of the parent and product ion yields shows the distinct dynamics of the individual ions.

For a pump–probe study of this inter-conversion, we used 102 μJ/pulse (i.e., 30% of our total laser intensity: not enough to ionize as it is below *I*_Th_) as the pump pulse to excite the DCPD to an electronic excited state (S_0_ → S_1_). After a variable delay, the probe laser pulse containing the remaining 70% of the total laser energy (i.e., 238 μJ/pulse) produces DCPD^+^ and CPD^+^, which are detected by the TOF mass spectrometer and in turn provides a snapshot of the decyclization reaction. The transient for a given mass is the ion signal versus delay time. Figure 7 shows the measured transients of the molecular ion and C_5_H_6_^+^. The overall nature of the transient measurements was reconfirmed at attenuated energies (as low as total laser energy of 200 μJ/pulse), when neither the pump (with 60 μJ/pulse) nor the probe (with 140 μJ/pulse) was above *I*_Th_.

**Figure 8:**
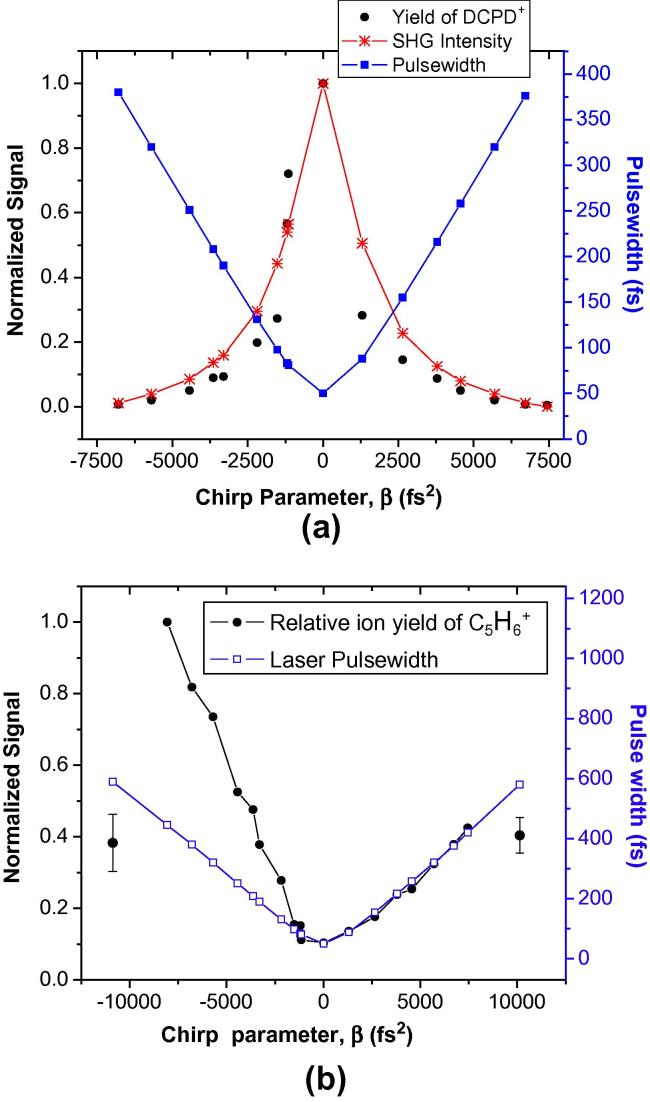
(a) Effect of chirp on the yield of DCPD^+^ is shown in comparison to the laser pulse width and the integrated SHG intensity generated by the pulse. (b) Effect of chirp on the relative yield of C_5_H_6_^+^ is shown as a function of the laser pulse width. Maxima in the measured error-bars are also plotted as representative cases for errors. At very large chirps, pulse stretching effect overwhelms as it substantially reduces the pulse intensity.

Under these experimental conditions, the observed dynamics has to occur where the probe laser induces the reactions resulting in further ionization [30]. The two-step decay model [26] was applied to explain the above-mentioned fragmentation of DCPD to CPD, shown in Figure 8a. The fitting of the rise and decay components of the transients were done by Matlab® programming using the curve fitting Levenberg–Marquardt algorithm. The best fit decay constants for the biexponential decay components of C_10_H_12_^+^ ion signal is *τ*_1_ = 35 fs and *τ*_2_ = 240 fs, while that for C_5_H_6_^+^ ion signal is *τ*_1_ = 36 fs and *τ*_2_ = 280 fs, respectively. These decay constants conform to the previously reported time constants of norbornene and norbornadiene [22], [23]. The transients of the reaction fragment C_5_H_6_^+^ are sufficiently different from that of the parent ion C_10_H_12_^+^ indicating that we are studying the distinct dynamics of the neutrals and not that of the parent ion fragmentation [24]. Applying laser control principles under such experimental circumstances also confirms that we are controlling the product yield of C_5_H_6_^+^, resulting from the photochemical reaction of DCPD.

Product yield distribution of a laser induced chemical reaction depends upon the coupling between the potential energy surfaces participating in the chemical reaction, as well as the characteristics of the interacting laser field, such as, its frequency, pulse duration, intensity, and chirp. Quantum mechanical wavepacket *Ψ*(*t*) produced through absorption of an ultrafast laser pulse is expressed as: *Ψ*(t) = $\sum_{n}a_{n}e^{-i2\pi E_{n}^{t/h}}$, where *E_n_* is the Eigen value and *φ_n_* is the Eigen functions of each level *n*, and the quantity *a_n_* is given by the Frank–Condon overlap between the initial and each final state *n*. The exponential part remains the same for all states when the when it is excited with transform limited pulses or unchirped pulses. However, using the chirped pulse excitation we can introduce different initial phases. Chirped pulse interaction, therefore, influences wave-packet shape and dynamics. This, in turn, changes the photochemical outcome and, as discussed earlier [14], is a chemical reaction control.

The phase (*φ* (*ω*)) of the laser pulse, centered at *ω_0_*, can be expressed as:

$\varphi (\omega )\approx \varphi (\omega_{0})+\frac{1}{1!}\frac{\partial \varphi }{\partial \omega }{|}_{\omega =\omega_{0}}(\omega -\omega_{0})+\frac{1}{2!}\frac{\partial^{2}\varphi }{\partial \omega^{2}}{|}_{\omega -\omega_{0}}(\omega -\omega_{0}{)}^{2}$. The second order term in the above expression $\beta =\frac{\partial^{2}\varphi }{\partial \omega^{2}}$ is responsible for group velocity dispersion. *β* is linear chirp coefficient (chirp parameter in the frequency domain) introduced by the compressor and is defined as the second derivative of spectral phase at the center frequency and can be calculated as: $\beta =\frac{\tau_{0}\sqrt{\tau^{2}-\tau_{0}^{2}}}{4\ln2}$, where *τ* is the pulse duration of the chirped laser pulse and *τ_0_* is the chirp-free pulse (transform limited pulse) [31]. This calculated value of chirp is within ±9%. Increasing or decreasing the distance between the two gratings in the compressor of our amplified laser produces the linearly chirped pulses. The pulse durations for different chirped pulses used are measured by intensity autocorrelation.

We recorded the comparative time of flight (TOF) mass spectra of DCPD using different amounts of linearly chirped pulse versus unchirped ultrafast laser pulses at a constant average energy of ∼200 μJ/pulse. The corresponding peaks in the mass spectra (C_5_H_6_^+^ and DCPD^+^, respectively) were compared by calculating their respective integrals under the ion peaks and normalizing them to that of the parent ion (DCPD^+^). The parent ion yield reduces in both the chirp directions symmetrically, which essentially follows the peak intensity of the laser pulse. This can also be confirmed through a measure of the integrated second-harmonic generated (SHG) intensity with 50 μm type-1 BBO crystal as a function of chirp. The short pulse favors the formation of the parent molecular ion as it can easily pluck out its electron. The sign of chirp has no role and yield of the parent ion is reduced in both the chirp direction symmetrically. Both SHG and DCPD^+^ ion yield is reduced symmetrically with respect to 0 fs^2^ (i.e., unchirped) as we increase the pulse duration in both the direction of chirp (Figure 8a). Very unlike the parent ion peak, the relative yield of C_5_H_6_^+^ (*m*/*z* = 66) arising out of the conversion product of DCPD is enhanced with increase in chirp in either direction of the linear chirp. However, the enhancement is found to be non-symmetric, favoring the negative chirp over positive chirps (Figure 8b).

Our results, in fact, show that the positive chirp effect is essentially the same as increasing the unmodulated pulse width. This can argued from the fact that this reaction occurs over 320 fs (biexponential fit timescales of pump–probe data), which indicates that we expect an increase in the relative yield of C_5_H_6_^+^ as the pulse width increases to ∼320 fs, either due to chirp or at lower bandwidths for unmodulated pulses [32]. However, the negatively chirped pulses favor the decyclization process over and above the pulse width correspondence, leading to an order of magnitude increase in the relative yield of C_5_H_6_^+^. This implies that the negative chirp observed enhancements are not due to pulse width effects but are dependent on the magnitude and sign of the linear chirp (Figure 8b).

Similar observations have been found earlier in the case of enhancement [14] of I_2_ elimination from CH_2_I_2_ and enhancement of multiphoton absorption in molecular iodine [33] by chirped pulse. Yakovlev et al. [33] were able to explain their observed results by quantum mechanical calculation which shows that wave packet following effect is one of the reasons behind the enhancement of three photon absorption with positively chirped pulse. Recently, Irimia et al. [34] also elucidated similar kind of control mechanism of CH_2_BrCl using velocity map photoelectron and ion imaging technique. Since potential energy surfaces involved in our particular chemical reaction is unknown, and no potential energy surface can also be calculated for this system with sufficient accuracy, an accurate description of the effect is not currently possible. However, it can be conjectured in analogy to the previous experiments that a red-blue sequence of photon interactions starts and ends at different points on the potential energy surface than does a blue-red sequence which could plausibly lead to the relatively high enhancement of negative chirp fragmentation due to a better overlapping time between the pump-dump or pump-pump pulses as compared to that of the transform-limited case [33], [35].

Though this may look like a plausible explanation, it is important to note that the reaction product in our particular case [27] arises from the ground state reaction coordinate. Only the pump-dump scheme would, therefore, lead eventually to the eventual product fragment ion. Consequently, all the discussions for the wavepacket overlap above [33], [35] has to essentially translate to a constructive overlapping time of the pump-dump pulses to result back to the ground potential energy state from the initially excited state potential. Suitably overlapping time between pump-dump pulses may promote the ground-state returned vibrationally hot dicyclopentadiene to yield the cyclopentadiene product preferentially, which may then be followed by a four-electron multiphoton ionization resulting in the ion-yields as observed. The initial photon mediated pump-dump process resulting in the generation of vibrationally hot ground-state precursor must be the critical initial condition of the reaction coordinate in order to explain the major effect of the negative versus positively chirped pulses. There is probably some effect of chirp on the 4-photon ionization process of the molecular ion as well as the conversion product as well. However, such effects would fail to bring about the observed distinction in behavior of dicyclopentadiene ion yield as compared to that of cyclopentadiene ion. Nevertheless, for very highly chirped pulses, the effect of the intensity decrease due to pulse width increase, in fact, overwhelms the chirp effects as can be seen from the reduction in the cyclopentadiene ion yield at the end points of experimental data plotted in Figure 8b. These points also have the largest error-bars among our experiments and hence we show them in the figure as the worst possible case.

Last but not the least, it is perhaps important to point out that the chirp effects are only distinct from the SHG processes when there is a reactive pathway involved in the process of the photo-fragment: thus, in our case, we find, as a function of laser chirp the DCPD^+^ formation process distinctively followed the SHG yield while the CPD^+^ formation deviated. This statement is further reconfirmed when we also recognize that there have been distinctive chirp effects in our photo-fragmentation studies of *n*-propyl benzene [16], a molecule complex enough to involve complex reaction pathways to form different fragment patterns in comparison to the photo-fragmentation of small molecules like methane [17], which result in only direct fragmentation products are always in perfect agreement to the corresponding SHG yields as a function of the laser chirp.

## 4 Conclusions

In summary, we have demonstrated the importance of phase structure of a femtosecond pulse in affecting a photochemical reaction of decyclization of DCPD, which occurs on an ultrafast timescale, as evident from our one color femtosecond pump–probe experiments here. Linearly chirped pulses enhance the reaction product only for the negative chirps, and the enhancement in the relative yield of the product is an order of magnitude as compared to that of the unmodulated or positively chirped pulses.
